# Key role of MEK/ERK pathway in sustaining tumorigenicity and in vitro radioresistance of embryonal rhabdomyosarcoma stem-like cell population

**DOI:** 10.1186/s12943-016-0501-y

**Published:** 2016-02-20

**Authors:** Carmela Ciccarelli, Francesca Vulcano, Luisa Milazzo, Giovanni Luca Gravina, Francesco Marampon, Giampiero Macioce, Adele Giampaolo, Vincenzo Tombolini, Virginia Di Paolo, Hamisa Jane Hassan, Bianca Maria Zani

**Affiliations:** Department of Biotechnological and Applied Clinical Sciences, University of L’Aquila, Via Vetoio, Coppito 2, 67100 L’Aquila, Italy; Department of Hematology, Oncology and Molecular Medicine, Istituto Superiore di Sanità, Rome, Italy; Department of Radiotherapy, University of Rome “Sapienza”, Rome, Italy; Department of Anatomy, Histology, Forensic Medicine and Orthopedic, Section of Histology, Sapienza University of Rome, Rome, Italy

## Abstract

**Background:**

The identification of signaling pathways that affect the cancer stem-like phenotype may provide insights into therapeutic targets for combating embryonal rhabdomyosarcoma. The aim of this study was to investigate the role of the MEK/ERK pathway in controlling the cancer stem-like phenotype using a model of rhabdospheres derived from the embryonal rhabdomyosarcoma cell line (RD).

**Methods:**

Rhabdospheres enriched in cancer stem like cells were obtained growing RD cells in non adherent condition in stem cell medium. Stem cell markers were evaluated by FACS analysis and immunoblotting. ERK1/2, myogenic markers, proteins of DNA repair and bone marrow X-linked kinase (BMX) expression were evaluated by immunoblotting analysis. Radiation was delivered using an x-6 MV photon linear accelerator. Xenografts were obtained in NOD/SCID mice by subcutaneously injection of rhabdosphere cells or cells pretreated with U0126 in stem cell medium.

**Results:**

MEK/ERK inhibitor U0126 dramatically prevented rhabdosphere formation and down-regulated stem cell markers CD133, CXCR4 and Nanog expression, but enhanced ALDH, MAPK phospho-active p38 and differentiative myogenic markers. By contrast, MAPK p38 inhibition accelerated rhabdosphere formation and enhanced phospho-active ERK1/2 and Nanog expression. RD cells, chronically treated with U0126 and then xeno-transplanted in NOD/SCID mice, delayed tumor development and reduced tumor mass when compared with tumor induced by rhabdosphere cells. U0126 intraperitoneal administration to mice bearing rhabdosphere-derived tumors inhibited tumor growth . The MEK/ERK pathway role in rhabdosphere radiosensitivity was investigated in vitro. Disassembly of rhabdospheres was induced by both radiation or U0126, and further enhanced by combined treatment. In U0126-treated rhabdospheres, the expression of the stem cell markers CD133 and CXCR4 decreased and dropped even more markedly following combined treatment. The expression of BMX, a negative regulator of apoptosis, also decreased following combined treatment, which suggests an increase in radiosensitivity of rhabdosphere cells.

**Conclusions:**

Our results indicate that the MEK/ERK pathway plays a prominent role in maintaining the stem-like phenotype of RD cells, their survival and their innate radioresistance.

Thus, therapeutic strategies that target cancer stem cells, which are resistant to traditional cancer therapies, may benefit from MEK/ERK inhibition combined with traditional radiotherapy, thereby providing a promising therapy for embryonal rhabdomyosarcoma.

**Electronic supplementary material:**

The online version of this article (doi:10.1186/s12943-016-0501-y) contains supplementary material, which is available to authorized users.

## Background

Rhabdomyosarcoma is the most common soft tissue tumor in childhood, accounting for more than half of all soft tissue sarcomas in children [1, 2]. The embryonal rhabdomyosarcoma subtype (ERMS) accounts for about 70 % of all rhabdomyosarcoma cases. In ERMS tumors, the Ras pathway is frequently mutated [3]. Dysregulation of the Ras pathway may be a crucial event in muscle precursor cells leading to ERMS fate, as described in mice models [4, 5].

Tumors contain a sub-population of cancer stem cells (CSCs) or cancer stem-like cells which are considered to be responsible for tumor initiation, propagation, invasiveness and metastasis [6, 7]. Owing to the lack of universal markers for the isolation and identification of CSCs, enrichment of CSCs from tumors or cell lines through a non-adhesive culture system has been adopted as a means of characterizing their partial stemness phenotype [8–10]. Several CSC markers have been identified in solid tumors including cell surface markers CD133, CD90, CD117, CXCR4 and CD166, soluble protein aldehyde dehydrogenase 1 (ALDH1), and transcription factor nanog [6, 11, 12]. In particular, CD133 has been identified as a central marker of ERMS CSC [13]. In stem cell (SC) medium, ERMS cell lines form spheres, named rhabdospheres, that are enriched in the CD133 positive population and have been shown to be more tumorigenic and more resistant to commonly used chemotherapies [13]. CXCR4, which plays an important role in chemotactic and invasive responses in several solid tumors, increases in ERMS spheres [14]. A high expression of CD133 in human ERMS samples also correlates with an unfavorable clinical outcome [13]. Moreover, ALDH1 has been reported to be a potential marker of CSCs in ERMS [15] and of muscle stem cells that spontaneously undergo myogenic differentiation [16], as well as a marker of rapid isolation of the human myogenic progenitors for cell therapy [17].

Signaling pathways in cancer stem cell biology are increasingly being used to investigate the mechanisms underlying the drug resistance, tumor relapse and dormant behavior exhibited by many tumors [18, 19]. The inhibition of EGFR-mediated MEK/ERK signaling impairs stem cell self-renewal and reduces the propagation of the DU145 prostate cell line [20]. Moreover, disruption of K-Ras or downstream signaling in colorectal cancer cell lines impairs CD133 expression [21].

One of the main indicators of the sensitivity of cancer cells to chemotherapeutic agents is believed to be apoptosis, particularly via the intrinsic mitochondrial cascade. Various integrated signals converge on BAK, an important effector of intrinsic apoptosis. BAK is negatively regulated by BMX, a tyrosine kinase, which associates with and phosphorylates BAK, thereby contributing to its inactivation [22]. BMX is often overexpressed in cancer cells to promote the survival of cancer.

It has been suggested in a previous work that MEK/ERK signalling is directly involved in the prevention of apoptosis [23]. The authors discussed the mechanism underlying BAK-mediated mitochondrial apoptosis and MEK/ERK-mediated inhibition of tyrosine phosphatase, which affects BAK phosphorylation and activation, thereby contributing to maintain cell survival [23].

Besides playing a role in the inhibition of apoptotic mechanisms, BMX is also required for maintenance of stem-like phenotypes in glioblastoma [24].

In ERMS, the main pathways involved in CSC survival and growth in the tumor environment have not yet been clearly defined. The MEK/ERK pathway has been shown to play a critical role in controlling cell growth, radioresistance and differentiative signals in the RD [25]. An interplay between ERKs and p38 mitogen-activated protein kinase (MAPK) has also been hypothesized [26].

In this study, inhibition of MEK/ERK signaling by U0126 reduces the size and tumorigenicity of the stem-like RD cell population. Furthermore, U0126 treatment enhances the inhibitory effect of radiation on stem-like rhabdomyosarcoma cells by favoring apoptosis. These findings highlight the potential advantage of using MEK/ERK inhibitor to target embryonal stem-like rhabdomyosarcoma cells.

## Methods

### Sphere culture, sphere formation assay, treatments and radiation exposure

Embryonal rhabdomyosarcoma cell lines, RD and TE671 (HTL97021), were procured from the American Type Culture Collection and Interlab Cell Line Collection, respectively.

Alveolar RH30 was obtained from DSMZ (Braunschweig, Germany). Sphere-forming cells were obtained as described [27]. Briefly, RD cells were cultured in anchorage-independent conditions (low attachment flasks or plates, Nunc) in SC-medium consisting in DMEM:F12 medium (Gibco-Invitrogen) with progesterone (2 μM), putresceine (10 μg/ml), sodium selenite (30nM), apo-transferrin (100 μg/ml) and insulin (50 mg/ml) (all from Sigma-Aldrich). Fresh human epidermal growth factor (20 ng/ml) and fibroblast growth factor (20 ng/ml) (PeproTech, London, UK) were added twice/week until cells formed floating spheres.

To evaluate the primary sphere formation, cells from sub-confluent (70–80 %) monolayer cultures were plated at a density of 100, 500 or 1000 cells in a 24-well culture plate (Corning Inc, Corning, NY, USA). For the sphere formation assay, the number of primary tumorspheres was counted. The primary spheres were mechanically dissociated and re-plated together with residual cell aggregates to obtain the second generation of spheres (Additional file 1: Figure S1).

MEK/ERK inhibitor U0126 (Promega, Madison, WI, USA) and MAPK p38 inhibitor SB203580 (Calbiochem, Nottingham, UK) were dissolved in dimethylsulfoxide (DMSO; Sigma-Aldrich) and used at the concentrations indicated. For a dose–response curve, RD cells, plated at a density of 1000 cell/well, as described above, were treated with varying concentrations of U0126 (1–20 μM) (3 wells per treatment) and spheres were counted. SB203580 was used at 2.5 μM, according to previous tests [26]. TE671 and RH30 were treated with 10, 20 or 40 μM U0126.

Radiation was delivered at room temperature using an x-6 MV photon linear accelerator, as previously described [28]. The total single dose of 4 Gy was delivered with a dose rate of 2 Gy/min using a source-to-surface distance (SSD) of 100 cm. A plate of Perspex thick 1.2 cm was positioned below the cell culture flasks in order to compensate for the build-up effect. Tumor cells were then irradiated placing the gantry angle at 180°. Non-irradiated controls were handled identically to the irradiated cells with the exception of the radiation exposure. The absorbed dose was measured using a Duplex dosimeter (PTW).

### Flow cytometer analysis

Stem cell markers in rhabdomyosarcoma cells were evaluated by staining with monoclonal antibodies conjugated with phycoerythrin (PE) anti–CD133 anti–CD90, anti–CXCR4, anti−CD105, and with allophycocyanin (APC) anti-CD117(all from BD Biosciences, Buccinasco, Italy).

Appropriate isotype controls for non-specific binding were used for each antibody. A minimum of 50,000 events were acquired for each sample by a flow cytometer (FACSCalibur, BD Biosciences) using CellQuest software (BD Biosciences) for data acquisition and analysis.

### Cell cycle analysis

A DNAcon3 kit (Dako, Glostrup, Denmark) was used for DNA staining. Briefly, 1 ml propidium iodide solution was added to each test tube containing dehydrated buffer mixture. After 10 min, cells were added to each tube and incubated at 4 °C for 1 h. Analysis was performed with FACScalibur, and the cell-cycle distribution was analyzed using Mod-Fit software (Verity Software House, Topsham, ME, USA).

### Aldefluor assay

The stem cell population expressing ALDH enzymatic activity was assessed by means of the Aldefluor™ kit (StemCell Technologies, Vancouver, BC, Canada), according to the manufacturer’s instructions. Briefly, 1 × 10^5^ cells were resuspended in Aldefluor assay buffer containing ALDH-substrate, and incubated for 45 min at 37 °C; a set of cells was stained using identical conditions with diethylaminobenzaldehyde, a specific ALDH inhibitor, as a negative control. Samples were analyzed by means of FACSCalibur, and the resulting fluorescence profiles were compared.

### Immunoblot analysis

Cells were lysed in Tris–HCl 10 mM pH 7.5, 1 % SDS containing phosphatase and protease inhibitors (Roche, Mannheim, Germany). Proteins were separated by SDS-polyacrylamide gel electrophoresis and transferred to a nitrocellulose membrane (Schleicher & Schuell, BioScience, Germany) by electroblotting. Immunoblotting was performed with the following antibodies: anti-Nanog, anti-ERK, anti-phospho-ERK1/2,anti- myogenin, anti-αtubulin, anti-GAPDH, anti-DNAPKcs, anti-Rad51, anti-BMX (all from SantaCruz Biotechnology, Santa Cruz, CA), anti-phospho-p38 (Cell Signaling Technology, Danvers, MA, USA) and anti- myosin heavy chain (MHC) (MF20 supernatant of hybridoma). Anti-mouse or anti-rabbit HRP-conjugated antibodies (Bethyl Laboratories Inc., Montgomery, TX, USA) were used for ECL (GE Health Life Sciences, Piscataway Township, NJ, USA) detection. Signals from protein bands were digitally acquired and quantified using the Chemidoc XRS system (BIORAD, Brossard, QC, Canada).

### Invasion assay

Invasion assay was used to assess the invasive potential of the cells, according to the standard protocol. Briefly, cells were plated in the upper chamber of a 24-well Transwell plate (8 μm pore size filter; Corning Inc., Corning, NY, USA) at a density of 80,000 cells/well in 200 μl of SC medium. 750 μl of SC medium containing 10 % FBS was added to the lower chamber as a chemoattractant, or SC medium alone as a negative control. After 24 h at 37 °C, non-invading cells were removed from the upper surface of inserts with a cotton swab and invaded cells were fixed with 4 % paraformaldehyde and stained. The number of cells that invaded the filter was counted using a bright-field microscope. Ten randomly selected fields were counted for each filter and the experiments were carried out twice in triplicates.

### Apoptosis assay

The Annexin/V-PI assay was carried out using the Annexin V-FITC Apoptosis Detection Kit (MERK Millipore). Rhabdospere RD cells were harvested and the pellets were immediately resuspended in the binding buffer provided. Cells were stained with 5 μl of FITC Annexin V and 5 μl of PI. The mixture was left to incubate at room temperature for 15 min and then was acquired by FACSCalibur (BD Biosciences) and analyzed using CellQuest software (BD Biosciences).

### NOD/SCID mice transplantation

NOD/SCID mice were bred and maintained under defined conditions at the Experimental Animal Welfare Sector of the Istituto Superiore di Sanità, Rome, Italy. All animal procedures complied with the European Community Directive on the welfare of experimental animals (Directive 2010/63/EU) upon approval of the protocol by the Institutional Animal Experimentation Committee. Equal numbers (2× 10^6^ cells) of adherent RD parental cells or rhabdosphere-derived cells suspended in 100 μl of phosphate buffered saline were subcutaneously injected into four- to six-week-old NOD/SCID female mice. Viability of the injected cells was confirmed by trypan blue (Sigma) staining prior to injection. Intraperitoneal injections of U0126 started when tumors reached a volume of 80–100 mm^3^. U0126 solution was prepared in DMSO as a stock solution of 10 mmol/L, and the amount of drug (25 μmol/Kg/mouse) to be injected into a set of mice was diluted with carrier solution (40 % DMSO in physiologic solution). The U0126 dose used here had previously been tested and found to be non-toxic in mice and to down-regulate ERK1/2 in tumors [25]. U0126 was administered 3 times per week. This protocol was chosen because full inhibition of ERK activation is guaranteed in vivo after 24 h and was documented after this time [25]. Four weeks after the beginning of treatment, the mice were killed by cervical dislocation and the tumors removed and weighed.

For U0126 pre-treatment, RD cells were cultured in SC medium in the presence of 10 μM U0126 for 15 days, followed by the transplantation of 2 × 10^6^ cells into the flank of NOD/SCID mice. Following cell injection, mice did not receive U0126 for the rest of the in vivo period. Four weeks after tumor appearance, the mice were killed by cervical dislocation and the tumors removed and weighed.

### Assessment of in vivo response to treatments

The effects on tumor growth of different treatments were evaluated by measuring the following: (1) tumor volume measured during and at the end of the experiment. Tumors were measured with a vernier caliper every 4 days and their volume, expressed in mm^3^, was calculated as length x (width)^2^/2. (2) Tumor weight measured at the end of experiment; (3) tumor progression, defined as an increase greater than 100 % of the tumor volume at the beginning (80–100 mm^3^) of the U0126 treatment delivered intraperitoneally; (4) time to progression. In the experiments in which the incidence of tumor development was studied, the occurrence of this event was defined as the appearance of a measurable (80–100 mm^3^) subcutaneous tumor lesion at the site of cell injection.

### Dissociation of tumor into single-cell suspension

For the FACS analysis, cells from xenograft tumors were obtained by means of the tissue dissociation protocol that combines mechanical and enzymatic approaches. Isolated tumor was minced with a sterile scalpel and digested with a solution of 1.5 mg/ml collagenase II (Gibco,) and 20 μg/ml DNAse I (Sigma) added to the tumor during mincing to facilitate tumor dissociation. After 2 h of incubation at 37 °C, cells were dissociated, washed and processed for FACS analysis.

### Statistical analysis

Continuous variables were summarized as the mean and S.D. or 95 % CI for the mean. Statistical comparisons between controls and treated groups were established by carrying out the Student’s t test for unpaired data (for two comparisons). Dichotomous variables were summarized by absolute and/or relative frequencies. For dichotomous variables, statistical comparisons between control and treated groups were performed by means of the exact Fisher’s test. The incidence of tumor development and tumor progression were analyzed by using Kaplan-Meier curves and Gehan’s generalized Wilcoxon test. Curves were compared by means of the log rank test and determination of the hazard ratio (HR). All the tests were two-sided and were determined by Monte Carlo significance. *P* values <0.05 were considered statistically significant. SPSS (statistical analysis software package, IBM Corp., Armonk, NY, USA) version 10.0 and StatDirect (version. 2.3.3., StatDirect Ltd, Altrincham, Manchester, UK) were used for the statistical analysis and graphic presentation.

## Results

### MEK/ERK inhibition affects the in vitro stem cell-like phenotype in embryonal, but not alveolar, rhabdomyosarcoma cell lines

**Fig 1:**
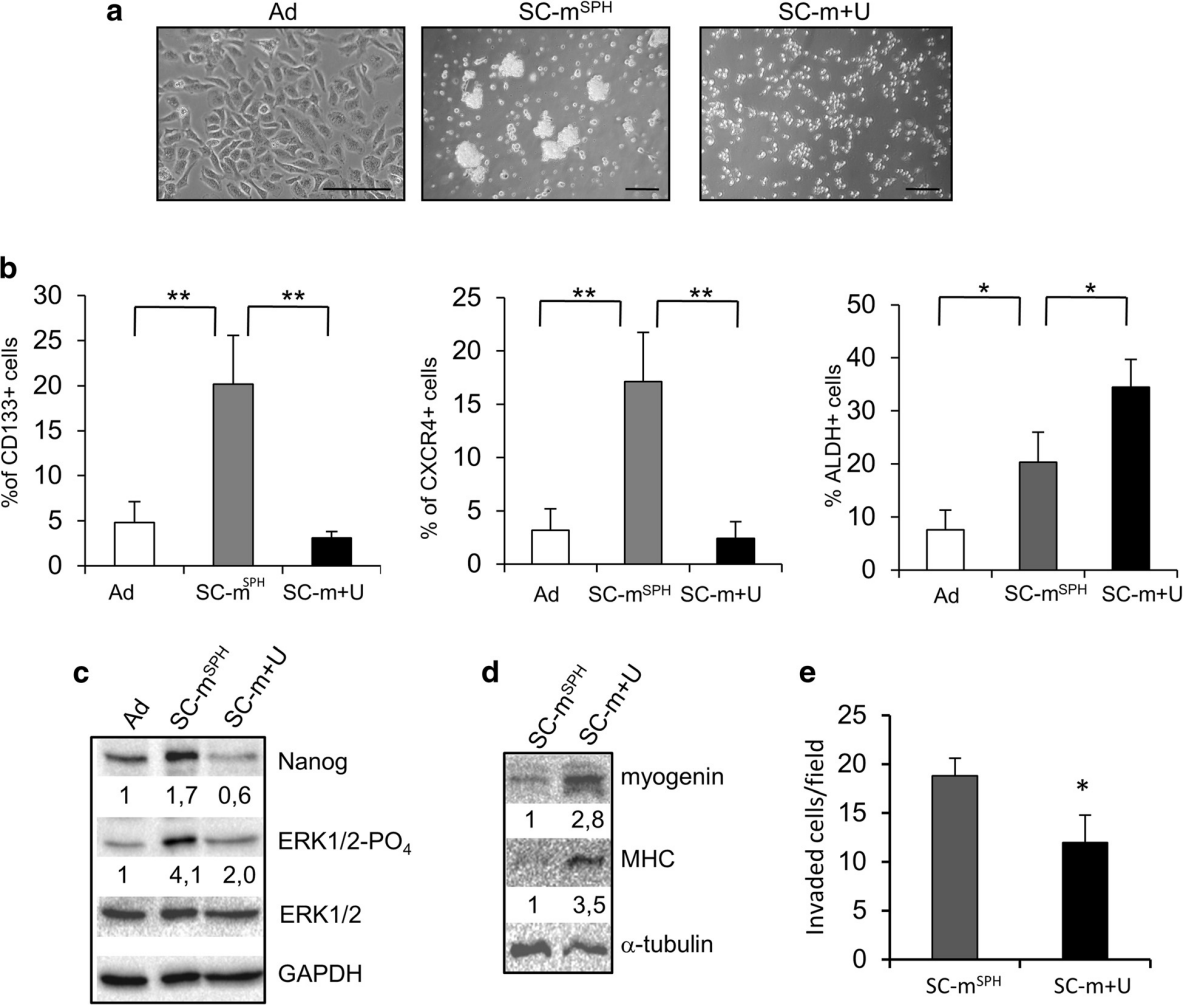
Expression of stem cell phenotype in RD cells and its inhibition by U0126. **a** Representative microphotographs of RD cells in adherent conditions (Ad) and in SC medium after 15 days of incubation in the absence (rhabdospheres, SC-m^**SPH**^) or in the presence of 10 μM U0126 (SC-m + U) (Bar = 100 μm). **b** Histograms of percentage of CD133, CXCR4 and ALDH positive cells determined by FACS analysis. Values represent the mean ± SD of 5 independent experiments. Statistical significance is indicated (**p < 0.01,*p < 0.05). **c**-**d** Western blot analysis of protein lysates from adherent RD cells (Ad) and RD in SC medium without (SC-m^**SPH**^) or with 10 μM U0126 (SC-m + U) at 15 days. **c** The expression levels of Nanog, ERK1/2-PO_4_ and ERK1/2 were analyzed. Protein bands were quantified by densitometry with respect to GAPDH. Representative experiment is shown. **d** The expression levels of myogenin and myosin heavy chain (MHC) were analyzed. Protein bands were quantified with respect to α-tubulin. Representative experiment is shown (out of three). **e** Cell invasion of RD in SC medium without (SC-m^**SPH**^) or with 10 μM U0126 (SC-m + U). Representative experiment is shown. Statistical significance is indicated (*p < 0.05)

RD cells grown in non-adherent conditions in the presence of SC-medium formed floating rhabdospheres in 10–15 days (Fig. 1a, middle panel). Rhabdosphere formation dependence on cell number was tested (Additional file 2: Figure S2).

The size of the stem-like population in rhabdospheres was evaluated using flow cytometry by assaying the percentage of the stem cell markers CD133, CXCR4 and ALDH (Fig. 1b). The percentage of CD133 positive cells was significantly higher in rhabdospheres than in adherent cells (20.2 % ± 5.4 vs 4.8 % ± 2.4; *p* < 0.01) and CXCR4 (17.1 % ± 4.6 vs 3.2 % ± 2.0; *p* < 0.01) and ALDH (7.6 % ± 3.7 vs 20.3 % ± 5.7; *p* <0.05) positive cells (Fig. 1b). No differences were observed in CD90, CD117, CD105 and CD166 expression (Additional file 3: Figure S3).

To ascertain whether the enriched RD stem-like population in rhabdospheres displayed a higher tumorigenic potential than the parental RD cell line, as has been reported elsewhere [13], adherent (1.97 % CD133 positive cells) or rhabdosphere cells (12.5 % CD133 positive cells) were injected into NOD/SCID mice (Additional file 4: Figure S4A). Tumors derived from rhabdosphere cells grew 1 month earlier and were larger than those induced by adherent cells (Additional file 4: Figure S4B), thereby suggesting that rhabdospheres were enriched in cancer stem-like cells characterized by a higher degree of in vivo tumorigenicity.

The role of the MEK/ERK pathway in maintaining the ERMS stem-like population phenotype was investigated. RD cells were cultured in SC-medium with or without U0126, a MEK/ERK inhibitor. 10 μM U0126 drastically reduced rhabdosphere formation (Fig. 1a). The inhibition of rhabdosphere formation was dose-dependent, 10 μM being the minimal dose displaying the maximum effect on rhabdosphere inhibition (Additional file 5: Figure S5). In SC-medium, U0126 treatment inhibited, by 85 %, the increase in the number of both CD133 (3.1 % ± 0.7 vs 20.2 % ± 5.4; *p* < 0.01) and CXCR4 (2.4 % ± 1.2 vs 17.1 % ± 4.6; *p* < 0.01) positive cells (Fig. 1b). By contrast, ALDH activity was further increased by U0126 (35.5 % ± 5.2 vs 20.2 % ± 5.4; *p* < 0.05) (Fig. 1b).

The expression of the stem cell marker Nanog in rhabdosphere cells, evaluated by immunoblot analysis, increased by about 70 % in comparison with adherent cells. In the presence of U0126, Nanog expression was inhibited by 65 % in comparison with untreated rhabdospheres (Fig. 1c). Phospho-active ERK1/2 levels were enhanced 4-fold in cells cultured in SC-medium in comparison with adherent cells, whereas U0126 treatment induced 50 % inhibition (Fig. 1c) that persisted up to 15 days (Additional file 6: Figure S6). Since MEK/ERK inhibition induces myogenic differentiation in RD cells, the expression of the differentiative markers myogenin and MHC was also analyzed in cells cultured in SC-medium in the presence of U0126. U0126 induced myogenin and MHC expression (Fig. 1d).

To investigate the effect of MEK/ERK on invasiveness, we probed rhabdosphere cells and U0126-treated cells in SC medium in an in vitro invasion assay. Invasiveness of MEK/ERK inhibited RD cells resulted decreased by 37 % in comparison with that of the rhabdosphere cells (Fig. 1e).

**Fig. 2:**
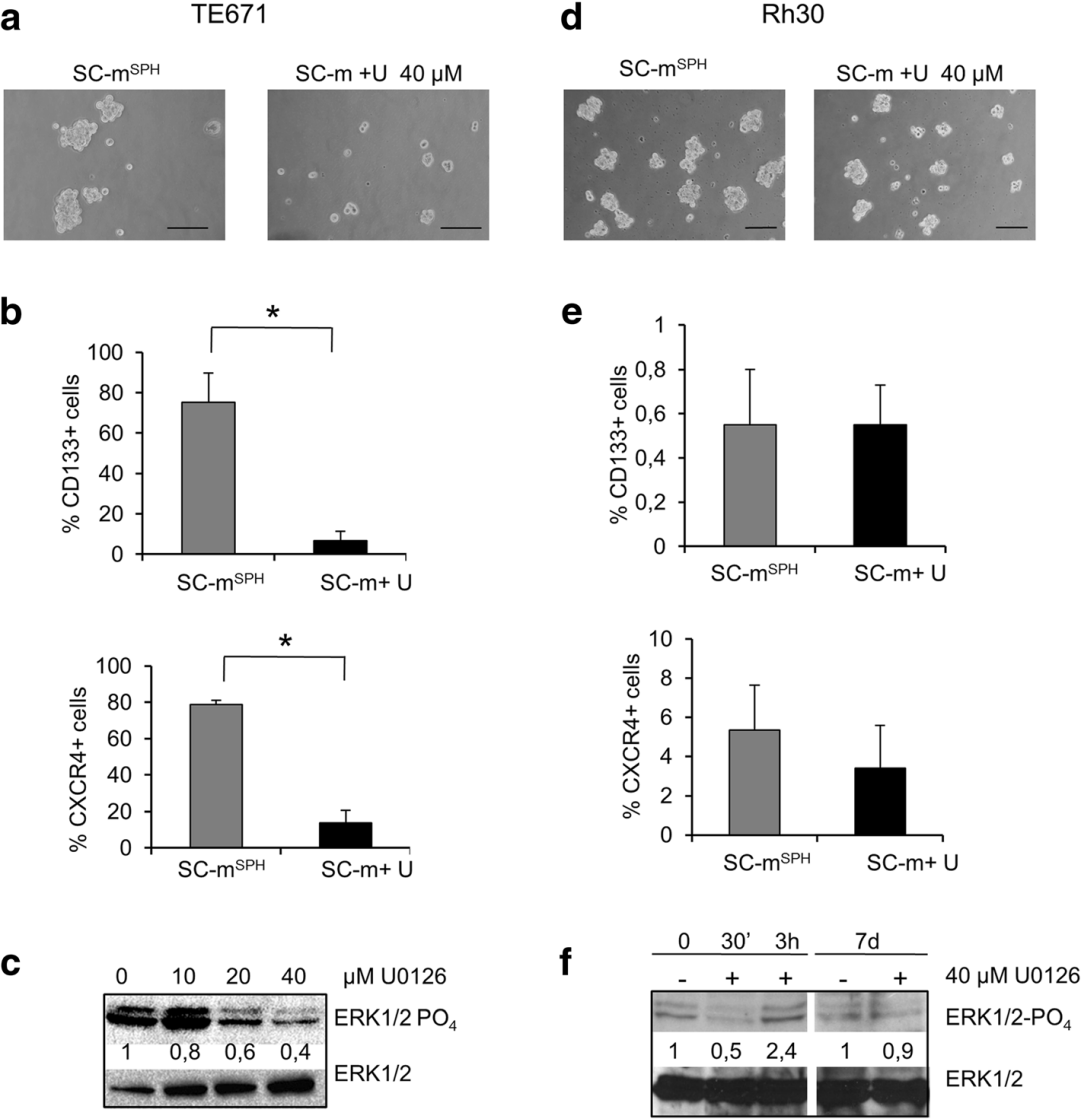
Expression of stem cell phenotype in other cell lines and effects of U0126. **a** and **d** Representative microphotographs of TE671 or RH30 cells in SC medium after 7 days of incubation in the absence (rhabdospheres, SC-m^SPH^) or in the presence of 40 μM U0126 (SC-m + U) (Bar = 100 μm). **b** and **e** Histograms of percentage of CD133 and CXCR4 positive cells determined by FACS analysis. Values represent the mean ± SD of 3 independent experiments. Statistical significance is indicated (*p < 0.05). **c** Western blot analysis of protein lysates from TE671 in SC medium without (SC-m^SPH^) or with different concentrations of U0126 (10–40 μM) (SC-m + U) at 7 days. **f** Western blot analysis of protein lysates from RH30 cells in SC medium without (SC-m^SPH^) or with 40 μM of U0126 (SC-m + U) at the times indicated. The expression levels of ERK1/2-PO4 and ERK1/2 were analyzed. ERK1/2-PO4 bands were quantified by densitometry and compared with ERK1/2

Rhabdosphere inhibition was also observed in TE671, another embryonal rhabdomyosarcoma cell line, though it required a higher concentration of U0126 (40 μM) (Fig. 2a).

In TE671, the stem cell markers CD133 and CXCR4 were highly expressed, while U0126 treatment strongly reduced the number of both CD133 (75 % ± 14.5 vs 6.7 % ± 4,5; *p* < 0.01) and CXCR4 (79 % ± 2.2 vs 14 % ± 6.8; *p* < 0.01) positive cells (Fig. 2b). Immunoblot analysis showed that ERK inhibition mirrors the inhibition of tumorsphere formation (Fig. 2c).

Since dysregulation of the Ras pathway is preferentially associated with ERMS and is responsible for the constitutive activation of MEK/ERK pathways, we included RH30, a rhabdomyosarcoma cell line that is negative for Ras mutations [29], to study the efficacy of MEK/ERK inhibition in cells lacking the constitutive activation of Ras/MEK/ERK pathways. Treatment with U0126 (10–40 μM) did not inhibit either rhabdosphere formation or CD133 and CXCR4 expression (Fig. 2d, e). Immunoblot analysis showed that ERK1/2 phosphorylation is transiently inhibited by U0126 treatment (30 min.), but recovers within 3 h (Fig. 2f).

### MEK/ERK and MAPK p38 pathway inhibition have opposite effects on RD stem-like phenotype

**Fig 3:**
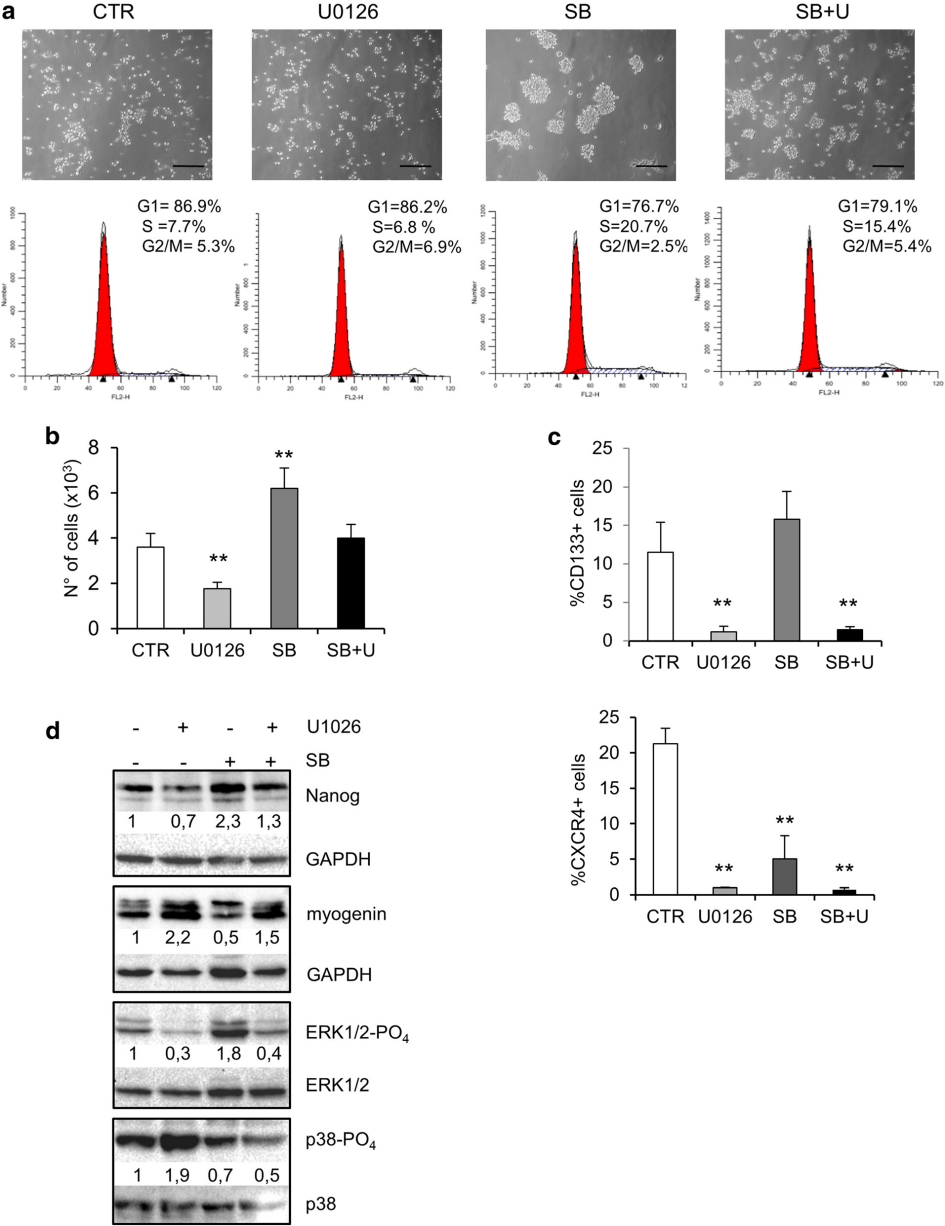
Effects of inhibition of p38 on sphere formation, stem and differentiative markers expression. RD cells were incubated in SC medium for 7 days (CTR) in the absence or in the presence of 2.5 μM SB203580 (SB) without or with 10 μM U0126 (SB + U). **a** Microphotographs (Bar = 100 μm) and cell cycle analysis of a representative experiment (out of three). **b** Histogram with cell numbers determined by cell counting. **c** Histograms of percentage of CD133 and CXCR4 positive cells determined by FACS analysis. Values represent the mean ± SD of 3 independent experiments. Statistical significance is indicated (**p < 0.01). **d** Western blot analysis of protein lysates. The expression levels of Nanog, ERK1/2-PO_4_, ERK1/2, myogenin, p38-PO_4_ and p38 were analyzed. Protein bands were quantified by densitometry with respect to GAPDH, ERK1/2

We showed that U0126 activates MAPK p38 in RD cells by inducing MEK/ERK inhibition [26]. To verify whether inhibition of RD stemness by U0126 is due to inhibition of phospho-active ERK1/2 and/or to MAPK p38 activation, we added SB203580 (SB), a MAPK p38-activity inhibitor, to the SC-medium at a concentration of 2.5 μM. MAPK p38 inhibition accelerated rhabdosphere formation, which appeared 5–7 days earlier than in cells cultured without SB (Fig. 3a). These effects were counteracted by SB and U0126 co-treatment (Fig. 3a).

The cell cycle analysis (Fig. 3a) and cell counting (Fig. 3b) showed the proliferative effects of SB treatment, which were attenuated by concomitant U0126 treatment (Figs. 3a, b). The analysis of CD133 positive population at day 7 did not reveal any major differences between RD grown in SC-medium in the absence and that grown in the presence of SB (Fig. 3c). Furthermore, SB treatment reduced the percentage of CXCR4 positive cells (5 % ± 3.3 vs 21.2 % ± 2.2) (Fig. 3c). In SB and U0126 co-treatment, the expression of CD133 and CXCR4 remained unchanged when compared with U0126 treatment alone (Fig. 3c). Nanog expression increased in the presence of SB on its own (Fig. 3d) but dropped markedly in the presence of both SB and U0126, thus suggesting a predominant effect of ERK inhibition. This hypothesis is supported by the analysis of the phospho-active ERK1/2 expression levels (Fig. 3d). In SB-treated cells, phospho-active ERK1/2 levels were considerably higher than in untreated RD cells, whereas when SB and U0126 were combined, phospho-active ERK1/2 levels were the same as those observed in U0126-treated RD cells.

In U0126-treated cells, MAPK phospho-active p38 levels were higher than in untreated RD cells and were inhibited in SB and U0126 treated cells. As expected, myogenin levels were increased by U0126 and inhibited by SB treatments. Nevertheless, myogenin levels in SB and U0126 treated cells remained above those observed in control untreated cells (Fig. 3d).

### U0126 impairs In vivo tumorigenicity of RD stem-like cells

**Fig. 4:**
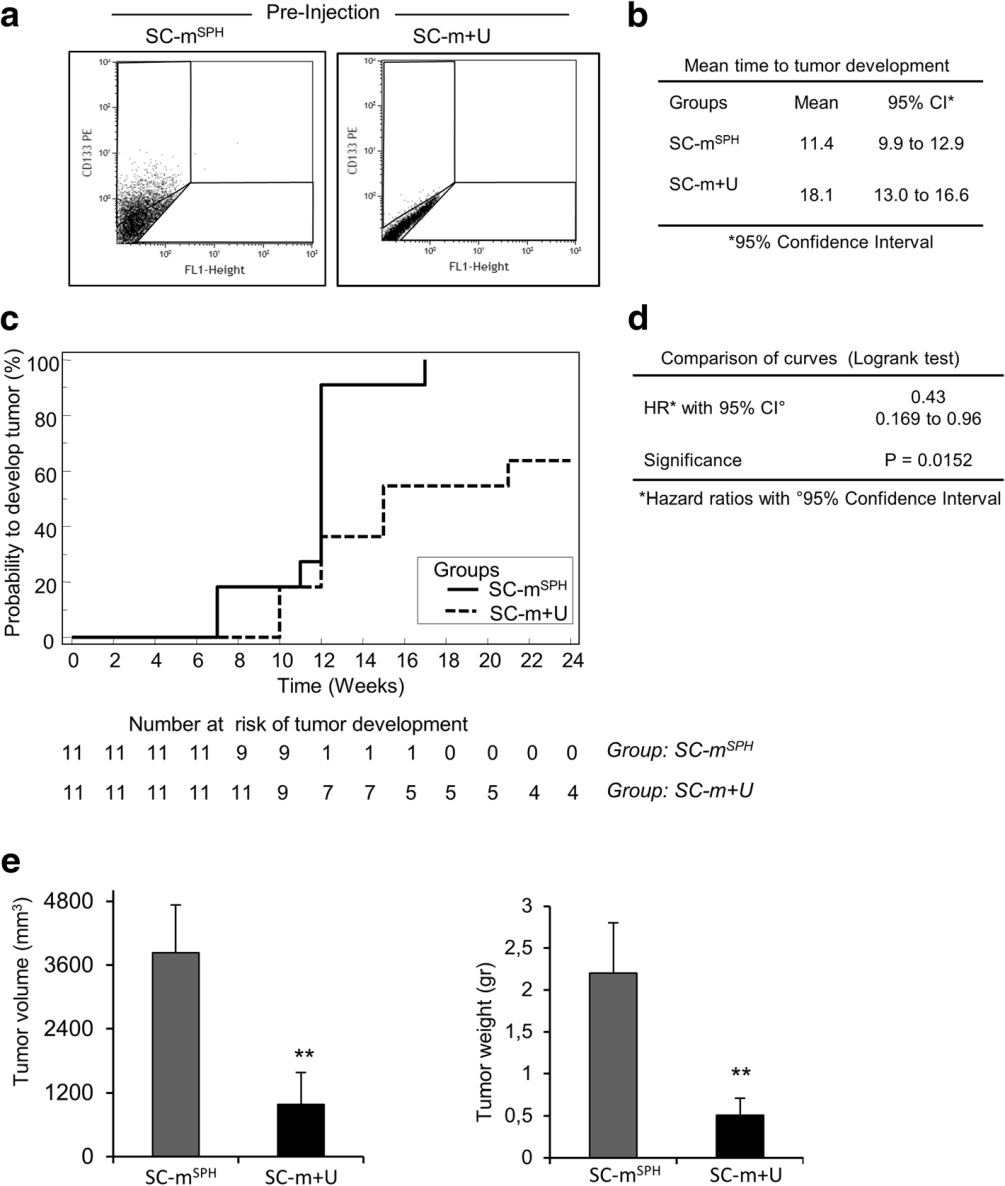
Delays of tumor development in xenografts from U0126 in vitro pre-treated cells. **a** FACS analysis of CD133 positive cells in rhabdosphere cells (SC-m^**SPH**^) and 10 μM U0126 treated cells in SC medium (SC-m + U) before subcutaneously injection. **b**-**e** Tumor development of xenografts from rhabdosphere cells (SC-m^**SPH**^) and U0126 treated cells in SC medium (SC-m + U). **b** Mean time to tumor development. **c** Analysis of tumor development by Kaplan-Meier. **d** Hazard Ratio (HR) with comparison of tumor development curves by Logrank test. **e** Tumor volume and tumor weight of xenografts at the end of the experiment (4 weeks after tumor development); bars represent mean ± S.D. Statistical significance is indicated (**p < 0.01)

The effects of MEK/ERK inhibition on tumorigenic potential were evaluated by inducing xenografts in NOD/SCID mice. For this purpose, RD cells in SC-medium were chronically (15 days) treated with U0126 before injection or left untreated. The percentage of CD133 positive cells in U0126 chronically-treated RD cells was lower (1.6 %) than in rhabdosphere untreated cells (27.3 %) (Fig. 4a). RD pre-treatment with U0126 postponed the mean time to tumor development by 6.7 weeks (Fig. 4b). These data are in agreement with those that emerge from the Kaplan-Meier curves, according to which tumor development occurred significantly more slowly in U0126 in vitro pre-treated cells (HR = 0.43; CI 95 % 0.169 to 0.96; *p* = 0.0152) than in rhabdosphere untreated cells (Fig. 4c). Indeed, the likelihood of developing a tumor in mice subcutaneously injected with U0126-pretreated RD cells was lower (57 %) (Figs. 4b, d). Interestingly, 4 out of the 11 (36.4 %) U0126 pre-treated RD cells had not developed tumors 24 weeks after cell injection, whereas all the animals injected with rhabdosphere cells had developed tumors after 17 weeks (Fig. 4c). A comparative analysis of tumor xenografts 4 weeks after their appearance showed that tumors induced by U0126 pre-treated RD cells were smaller than those induced by rhabdosphere cells (Fig. 4e).

**Fig. 5:**
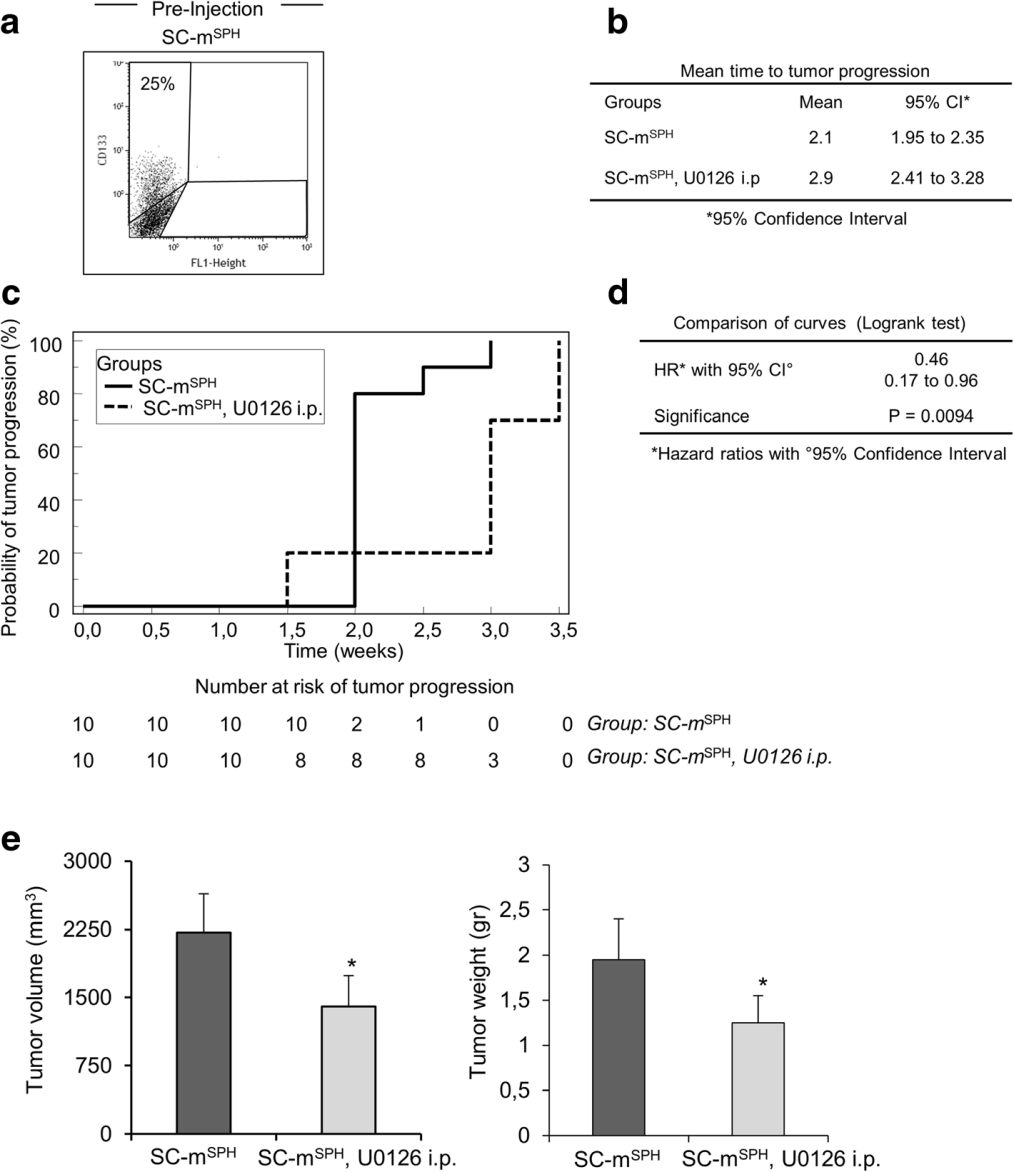
U0126 inhibits the growth of rhabospheres-derived xenografts. **a** FACS analysis of CD133 positive cells in rhabdosphere cells (SC-m^**SPH**^) before subcutaneously injection. **b-e** Mice bearing tumor xenografts derived from rhabdosphere cells were treated with vehicle (group: SC-m^**SPH**^) or with U0126 by intraperitoneal injection at 25 μmol/kg/mouse (group: SC-m^**SPH**^, U0126 i.p.); tumor size was assessed every 4 days. **b** Mean time of tumor progression. **c** Analysis of tumor progression by Kaplan-Meier curves. **d** Hazard Ratio (HR) with comparison of growth curves by Logrank test. **e** tumor volume and tumor weight at the end of the experiment (4 weeks of treatment). Bars represent mean ± S.D. Statistical significance is indicated (*p < 0.05)

The in vivo effect of MEK/ERK inhibition was also assayed by treating mice bearing xenografts induced by rhabdosphere cells (25 % CD133 positive cells) (Fig. 5a) with an intraperitoneal injection of 25 μmoles/kg of U0126 when the tumor mass reached a volume of between 80 and 100 mm^3^.

The mean time to tumor progression was delayed by about 0.8 weeks in the group of mice treated with U0126 compared with mice with rhabdosphere tumors (Figs. 5b, c). All the mice treated with U0126 displayed tumor progression within 3.5 weeks of reaching a volume of 80–100 mm^3^. Tumor progression in the rhabdosphere group was instead observed within 2.9 weeks of reaching a volume of 80–100 mm^3^. Mice treated with U0126 were less likely (54 %) to develop tumors (HR = 0.46; CI 95 % 0.17 to 0.96; *p* = 0.0094) (Fig. 5d). Tumor analysis 4 weeks after U0126 treatment showed that tumor xenografts from U0126-treated mice were smaller than those from control mice (Fig. 5e).

**Fig. 6:**
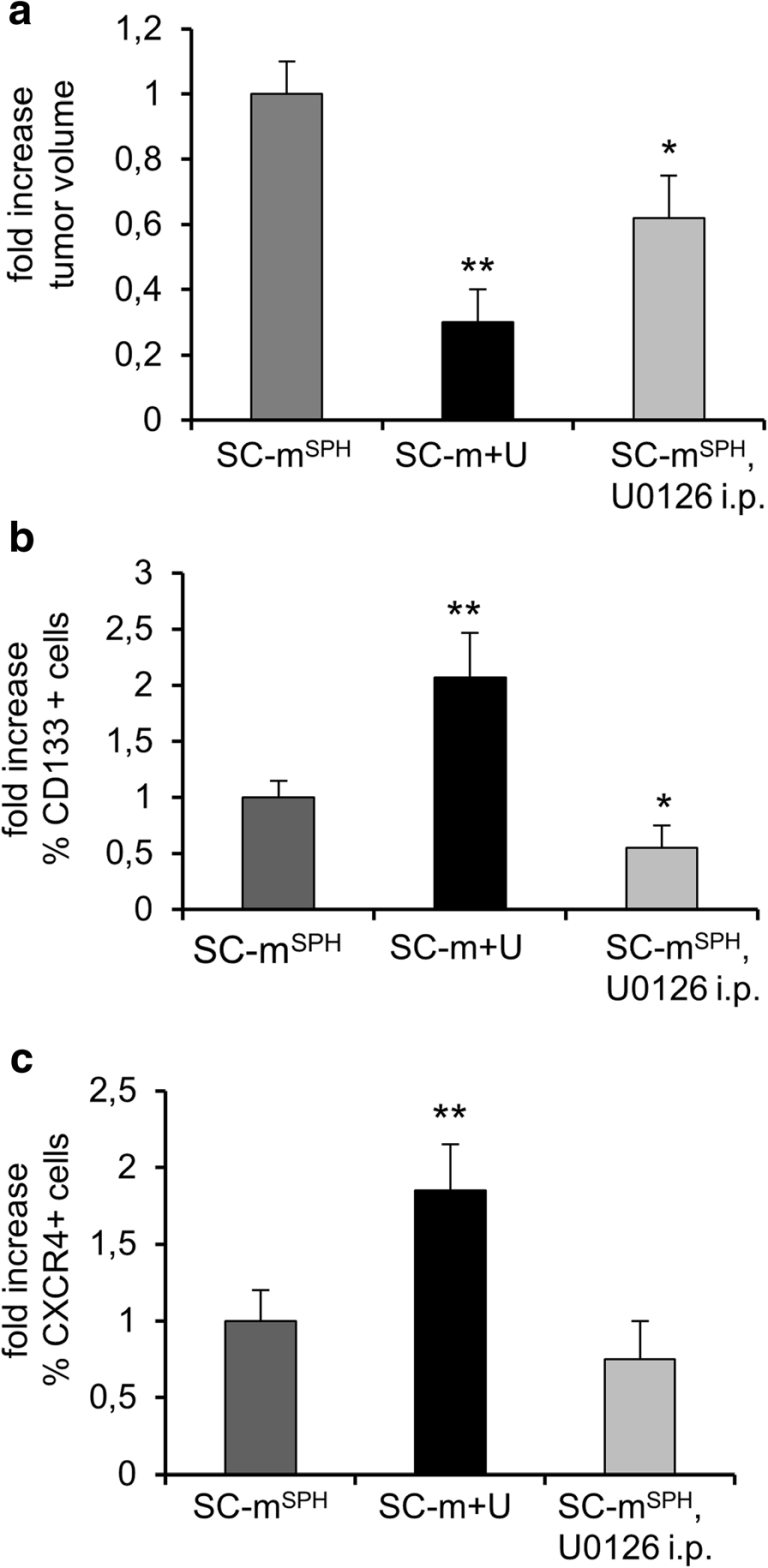
Fold variation of volume and stem cell markers expression in tumor xenografts. **a** Tumor xenografts volume from rhabdosphere cells (SC-m^**SPH**^), U0126 treated cells in SC medium (SC-m + U) and rhabdosphere cells after U0126 intraperitoneal injection (SC-m^**SPH**^, U0126 i.p.) at the end point (4 weeks after tumor development). **b** CD133-positive cells and **c** CXCR4 positive cells after digestion into single cell suspension of tumor xenografts (*n* = 3, **p < 0.01, *p < 0.05)

Comparing results of the two in vivo experiments, the greatest reduction in tumor volumes was observed in xenografts derived from U0126 pre-treated cells (Fig. 6a). We then compared CD133 and CXCR4 positive populations in dissociated xenografts from U0126-pretreated cells and intraperitoneal U0126-treated mice. The results show a significant increase (2 fold) in the size of the CD133 positive population in tumors induced by U0126 pre-treated RD cells compared with those induced by rhabdosphere cells. By contrast, the size of the CD133 positive population in dissociated xenograft cells from intraperitoneal U0126-treated mice was smaller (0.45 fold) than in those induced in mice treated with vehicle alone (Fig. 6b). A significant increase (1.8 fold) was observed in the size of the CXCR4 positive population in xenografts derived from U0126-pretreated RD cells (Fig. 6c), whereas a non-significant decrease was observed in the size of the CXCR4 positive population in xenografts derived from intraperitoneal U0126-treated mice (Fig. 6c).

### Effect of U0126 and radiation treatments in rhabdosphere maintenance and apoptosis

**Fig. 7:**
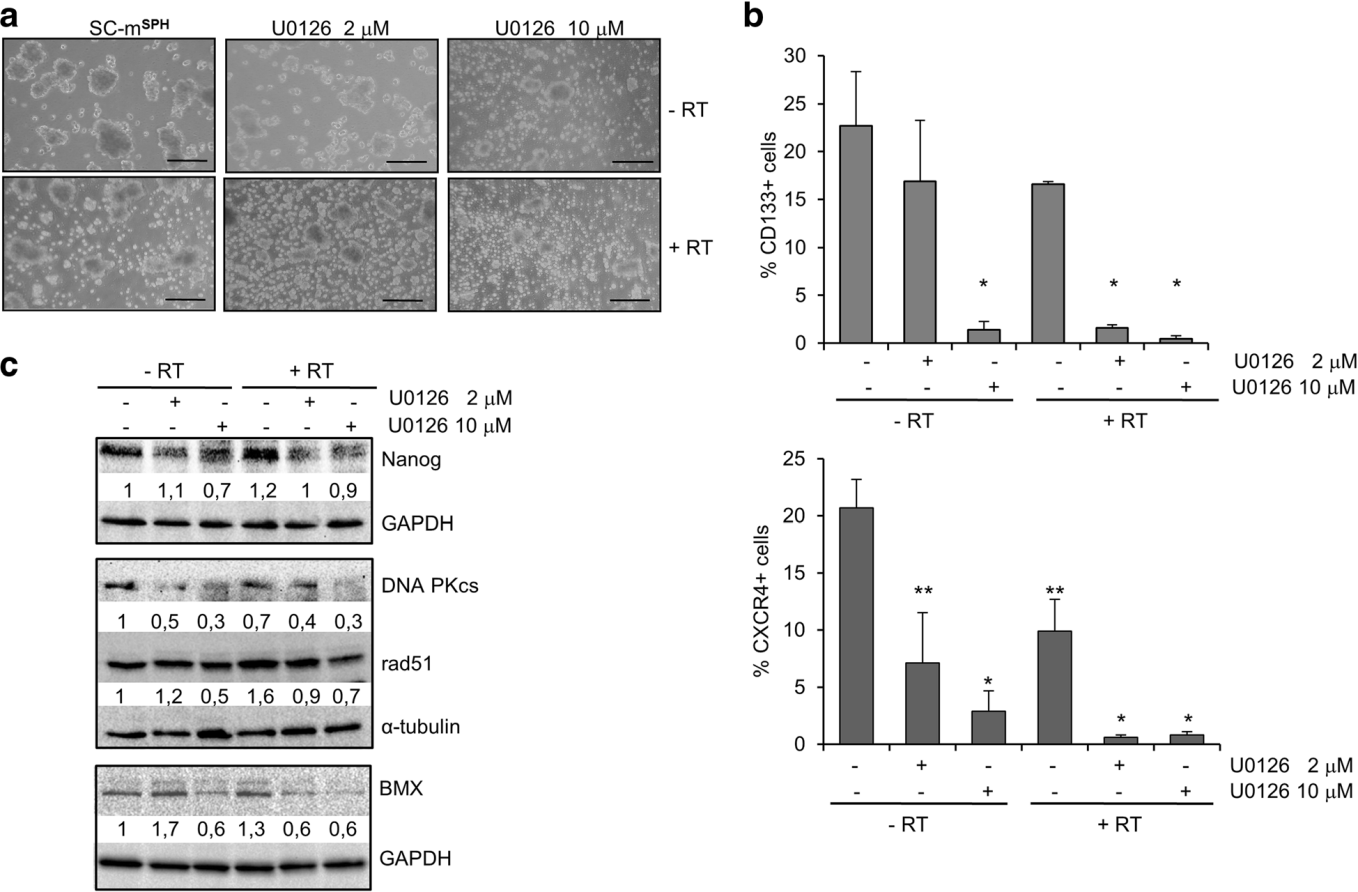
Effects of radiation on rhabdosphere cells and in combination with U0126. Radiation (RT) or/and 2 or 10 μM U0126 were provided after spheres formation (SC-m^**SPH**^). Samples were analyzed after 6 days. **a** Representative microphotographs. **b** Histograms of CD133 and CXCR4 positive cells determined by FACS analysis. Values represent the mean ± S.D. of 3 independent experiments (**p < 0.01, *p < 0.05). **c** Western blot analysis of Nanog, DNA-PKcs, Rad51 and BMX in protein lysates. Protein bands were quantified by densitometry with respect to GAPDH or α-tubulin. A representative experiment is shown

The rationale of rhabdosphere treatment with U0126 and/or radiation lies in the possibility it offers of targeting an enriched stem-like cell population. The use of two concentrations of U0126 allowed us to assess the minimal dose of inhibitor required to obtain the maximal effects when combined with radiotherapy. Treatment with 10 μM U0126 led to a clear disassembly of cells from rhabdospheres. Less evident disassembly was observed using a reduced U0126 concentration (2 μM) (Fig. 7a). Combined U0126 (2 or 10 μM) and radiation (4Gy) treatment enhanced the effect of sphere disassembly, further reducing the number and size of the spheres (Fig. 7a).

The analysis of the stem cell markers showed that either 2 μM U0126 treatment or radiation treatment slightly reduced the expression of CD133 and more markedly reduced that of CXCR4 (Fig. 7b). By contrast, combined treatment greatly reduced the expression of both stem cell markers. CD133 was reduced 10 fold (16.9 % ± 6.4 vs 1.6 % ± 0.3) and CXCR4 11 fold (7.1 % ± 4.4 vs 0.6 % ± 0.2). Treatment with 10 μM U0126 alone markedly reduced both CD133 and CXCR4 (1.4 ± 0.8 and 2.9 ± 1.8, respectively), and to an even greater extent when combined with radiation (0.45 % ± 0.3 and 0.8 % ± 0.3, respectively) (Fig. 7b).

We also analyzed the expression of Nanog and components of DNA repair machinery, such as Rad51 and DNA-PKcs, in rhabdospheres.

The results show that Nanog expression levels were unchanged following treatment with 2 μM U0126 and were reduced following treatment with 10 μM U0126. Nanog expression was increased slightly by radiation, but was inhibited by either concentration when combined with radiation (Fig. 7c).

The expression levels of DNA-PKcs were reduced by both concentrations of U0126 regardless of the presence or absence of radiation, the degree of inhibition being more pronounced in Rad51 expression following combined radiation and 10 μM U0126 treatment (Fig. 7c).

Since BMX may help to protect cells from apoptosis induced by radiation [30], we analyzed BMX expression in rhabdosphere after radiation in the presence or absence of U0126. The result shows that BMX expression is down-regulated at an even lower concentration (2 μM) of U0126 when combined with radiation, as demonstrated by the analysis of apoptotic cells (Additional file 7: Figure S7).

## Discussion

Cancer stem cell research is becoming increasingly important in the investigation of the development, spread, resistance to chemo- and radio-therapy and relapse of cancer.

We previously demonstrated, both in vitro and in vivo, the responsiveness of the RD cell line to MEK/ERK inhibition, which induces growth arrest, myogenic differentiation, radiotherapy sensitization and tumor growth impairment [25, 28]. On the basis of these data, we decided to assess the contribution of the MEK/ERK pathway in controlling the cancer stem-like compartment in the ERMS cell system.

It is generally agreed that tumorspheres enriched in cancer stem-like cells are highly tumorigenic [13, 27, 31, 32]. By culturing RD cells in SC-medium, we obtained rhabdospheres enriched in positive CD133, CXCR4, ALDH and Nanog stem-like cells that are highly tumorigenic in vivo. These results are consistent with those reported in previous studies [13–15].

Since Ras/ERK is an upstream pathway of CD133 expression [21, 33], the use of U0126, a MEK/ERK inhibitor, proved useful to study the dependence of a stem cell-like population on the MEK/ERK pathway. We used U0126 to demonstrate, for the first time, the critical contribution made by MEK/ERK signaling to the cancer stem-like phenotype in the RD cell line. Indeed, sphere formation is inhibited by U0126, in a dose-dependent manner, in the embryonal RD cell line though not in the Ras negative alveolar RH30 cell line. The RH30 cell line does not exhibit persistent ERK inhibition by U0126 whereas it does exhibit very low levels of CD133, thus suggesting that other pathways underlie the alveolar stem cell-like phenotype.

In rhabdospheres derived from RD cells, the stem cell markers CD133, CXCR4 and Nanog were enhanced and were dramatically inhibited by U0126 treatment, whereas ALDH activity was increased by MEK/ERK inhibition. This finding is in agreement with a recent report showing that high ALDH1 activity is related to the myogenic potential of muscle precursors [34]. It has also been demonstrated that the induction of myogenic differentiation occurs spontaneously in myogenic precursors that highly express ALDH1 [16]. The increased ALDH activity observed under MEK/ERK inhibition in the system we adopted may indeed be related to myogenic differentiation. In this regard, the differentiative markers myogenin and MHC are enhanced by the U0126 treatment of RD cells in SC-medium. We may therefore speculate that MEK/ERK pathway inhibition induces molecular reprogramming, which rescues the myogenic precursor phenotype in rhabdomyosarcoma cancer stem-like cells.

Together these data strongly suggest that the differentiation boost resulting from MEK/ERK inhibition turns off cancer stem-like phenotype expression.

Published studies by us and other authors have shown that MAPK p38 plays a pivotal role in myogenic differentiation [26, 35]. Here we found that inhibition of the differentiative action of MAPK p38 significantly enhances the expression of Nanog and phospho-active ERK1/2, correlates with an increased S phase of the cell cycle and accelerates sphere formation. The size of the CD133 positive cell population was not affected markedly under MAPK p38 inhibition in this study, and that of the CXCR4 positive population actually decreased. By contrast, Nanog and phospho-active ERK1/2 were significantly enhanced, thereby suggesting that they play a major role in rhabdosphere formation. These results agree with those recently reported on the role of Nanog in ERMS as an inducer of sphere formation, as an important gene for tumor promoting properties and as a prognostic marker for ERMS patients [36].

The positive effects of MAPK p38 inhibition on the stem-like phenotype are reverted by MEK/ERK inhibition when treatment with inhibitors is concomitant, thereby demonstrating that chronic MEK/ERK inhibition strongly impairs the stem-like phenotype in embryonal rhabdomyosarcoma.

The relevance of the active MEK/ERK pathway in cancer stem-like cells with tumor initiating properties is demonstrated by the significant delay in tumor development (11.4 vs 18.1 weeks) and reduced tumor size displayed by xeno-transplanted RD cells pre-cultured in the presence of the MEK/ERK inhibitor. It is noteworthy that our data showing that rhabdospheres express high ALDH activity, which is further increased by U0126 treatment, are only partially in agreement with those of other authors [15], who reported that ALDH1 is a marker of cancer stem cells in ERMS. This suggests that ALDH activity is sensitive to induced signaling. Indeed, U0126-treated cells that strongly express ALDH do not appear to play a role in early tumor initiation and the development of tumor masses, which would be expected to be larger than those of untreated rhabdospheres. The fact that tumor development is delayed and the tumors themselves are smaller may mean that the subpopulation that expresses a high degree of ALDH activity does not contribute to the tumorigenicity of cancer stem-like cells if the active ERK pathway is absent but undertakes the myogenic precursor program. This hypothesis is supported by the low expression level of CD133 and CXCR4 in U0126-pre-treated cells and correlates strongly with the delay in tumor development that occurred without any further U0126 being added. It is noteworthy that, at the end point, the number of CD133 and CXCR4 positive cells in this tumor population was two folds that in xenografts induced by rhabdospheres. This finding appears to be in contrast to the delay in tumor development and warrants further investigation. Moreover, the intraperitoneal U0126 treatment of mice xeno-transplanted with rhabdosphere cells inhibits tumor growth by about 50 %. The responsiveness of rhabdosphere-derived tumors to the MEK/ERK inhibitor in developing xenografts might be consequent to the reduction in the size of the CD133 population even in in vivo conditions. On the basis of all these in vivo data, continuous treatment with the MEK/ERK inhibitor might help to maintain low levels of CD133 and CXCR4 positive cells.

Following Ras activation, the MAPK pathway has been reported to contribute to the invasive potential of cancer cells [37]. Other authors have demonstrated that increased Caveolin 1 expression enhances ERK pathway activation and potentiates invasiveness of RD cells [38]. Furthermore, it is worth recalling data from others [39–41] that suggest the role of CD133 and CXCR4 content in sustaining high metastatic capacity in in vitro and in vivo model of some tumor types. However, the metastatic activity of cancer stem cells is a multistep process, that includes the invasiveness, but is not completely performable in vitro. The reduced invasion potential of U0126-treated cells in SC medium compared with rhabdosphere cells is in keeping with the reduced expression level of CXCR4 and CD133 in U0126-treated cells. Our result indicates that in RD cancer like stem cells invasion potential is a property that depends on ERK pathway.

Targeting MEK/ERK pathway to reduce the chemo- and radio-resistant CD133 positive cell population [13] may have important implications in the treatment of ERMS.

Indeed, the radioresistance phenotype of cancer stem cells might be the cause of cancer relapse [42, 43]. The reduction of CD133 positive population might have a beneficial effect on radiation efficacy given that in some cases in CD133 positive cells active ERKs is enhanced by radiation [44].

A combination of radiotherapy and chemotherapy is one approach currently being used to treat rhabdomyosarcoma. Within this context, the rationale underlying the treatment of rhabdospheres using U0126 was the hypothesis that MEK/ERK inhibition enhances radiosensitivity in the presence of an enriched cancer stem-like population. Radiation or U0126 treatment on their own modify the integrity of rhabdospheres, alter the percentage of stem cell markers and reduce the DNA machinery components levels, though radiation alone is less effective. Combined therapy induces a more pronounced dismantling of rhabdospheres and inhibits the expression of stem cell markers and Rad51. Bearing in mind that Rad51 expression is highly sensitive to MEK/ERK inhibitor [45], the markedly reduced levels of Rad51 and DNA-PKcs observed in U0126-treated rhabdospheres indicate that MEK/ERK inhibition impairs DNA repair mechanisms, thereby rendering RD cells more sensitive to radiation. The ability of MEK/ERK pathways to orchestrate the complex mechanism of survival in tumor cells, including resistance to radiation, is also demonstrated here by the MEK inhibitor-mediated down-regulation of BMX, whose absence is known to relieve cells from the negative regulation of apoptosis [22]. Therefore, the MEK/ERK-dependent inhibition of BMX expression [23] may be involved in the enhanced sensitivity of RD cells to radiation.

## Conclusions

Our results indicate that the MEK/ERK pathway plays a prominent role in maintaining the stem-like phenotype of RD cells. Furthermore, the MEK/ERK pathway inhibition makes RD cells more sensitive to radiation.

In conclusion, therapeutic strategies aimed at targeting cancer stem cells that are resistant to traditional cancer therapies may benefit from MEK/ERK inhibition combined with radiotherapy, and thus offer a promising therapy for embryonal rhabdomyosarcoma.

## Additional files

Additional file 1: Figure S1. CD133 positive population in primary (first generation) and secondary (second generation) rhabdospheres. CD133 positive cells have been determined by FACS. No major differences in size of CD133 positive population were detected between the two generation of rhabdospheres. (TIF 395 kb) Additional file 2: Figure S2. Histogram of rhabdospheres formation dependent on number of cells. RD seeded at 500 or 1,000 cells/well in SC-medium (three replicates/sample). Rhabdosphere were counted at indicated times. The sphere numbers are displayed as mean ± S.D. (TIF 122 kb) Additional file 3: Figure S3. Results of FACS analysis of the levels of CD90, CD117 and CD105 positive populations, which remain unchanged in SC-medium with respect to adherent condition. The percentage is displayed as mean ± S.D. of 3 experiments. (TIF 139 kb) Additional file 4: Figure S4. Tumorigenic assay of rhabdosphere cells. A) FACS analysis of CD133 positive cells in adherent and in rhabdosphere cells (SC-m^**SPH**^) before subcoutaneously injection (pre-injection); B) histogram showing the tumor development and volumes of xenografts from adherent (Ad) or rhabdosphere cells (SC-m^**SPH**^), (*n* = 6, **p* > 0,05). (TIF 341 kb) Additional file 5: Figure S5. U0126 dose/response of rhabdospheres inhibition. Histogram of two independent experiments of RD cells seeded in SC-medium (1,000 cells/well, three replicates per treatment) without or with indicated concentration of U0126. At13th day 1/3 of volume of fresh SC-medium containing the indicated U0126 concentration was added. Rhabdospheres were counted at indicated times. The sphere numbers are displayed as mean ± S.D. (TIF 122 kb) Additional file 6: Figure S6. Persistent phospho-ERK1/2 (ERK1/2-P) down regulation by U0126. Western blot analysis of protein lysates from RD in SC-medium without or with 10 μM U0126 at indicated times. The expression of ERK1/2-P was analysed and quantified by densitometry with respect to ERK/1/2. Representative experiment is shown. (TIF 385 kb) Additional file 7: Figure S7. Radiation (RT) or/and 2 μM U0126 were provided after RD rhabdophere spheres formation. Analysis of cell death by flow cytometric detection of Annexin-V staining of samples analyzed after 6 hours of treatment. (TIF 78 kb)

## Abbreviations

ALDH: aldehyde dehydrogenase
CSCs: cancer stem cells
ERMS: embryonal rhabdomyosarcoma subtype
MAPK: mitogen-activated protein kinase
MHC: myosin heavy chain
RD: embryonal rhabdomyosarcoma cell line
SB: SB203580
SC: stem cell
