# Supplementary figures and images for: Key role of MEK/ERK pathway in sustaining tumorigenicity and in vitro radioresistance of embryonal rhabdomyosarcoma stem-like cell population
Source: Mol Cancer. 2016 Feb 20;15:16. doi: 10.1186/s12943-016-0501-y (PMC4761200; doi:10.1186/s12943-016-0501-y)

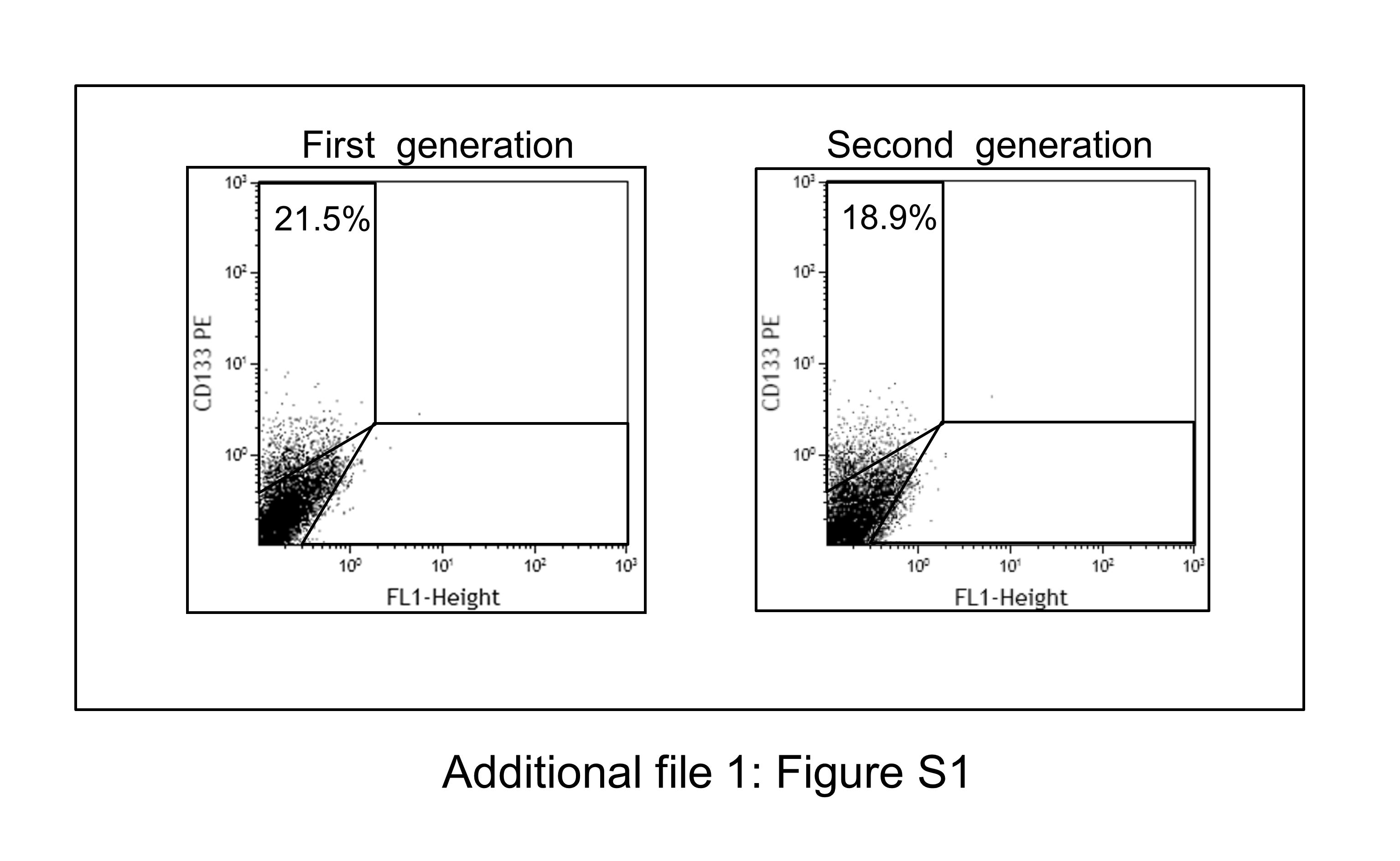

Supplement: Additional file 1: Figure S1. — CD133 positive population in primary (first generation) and secondary (second generation) rhabdospheres. CD133 positive cells have been determined by FACS. No major differences in size of CD133 positive population were detected between the two generation of rhabdospheres. (TIF 395 kb) [file 12943_2016_501_MOESM1_ESM.tif]

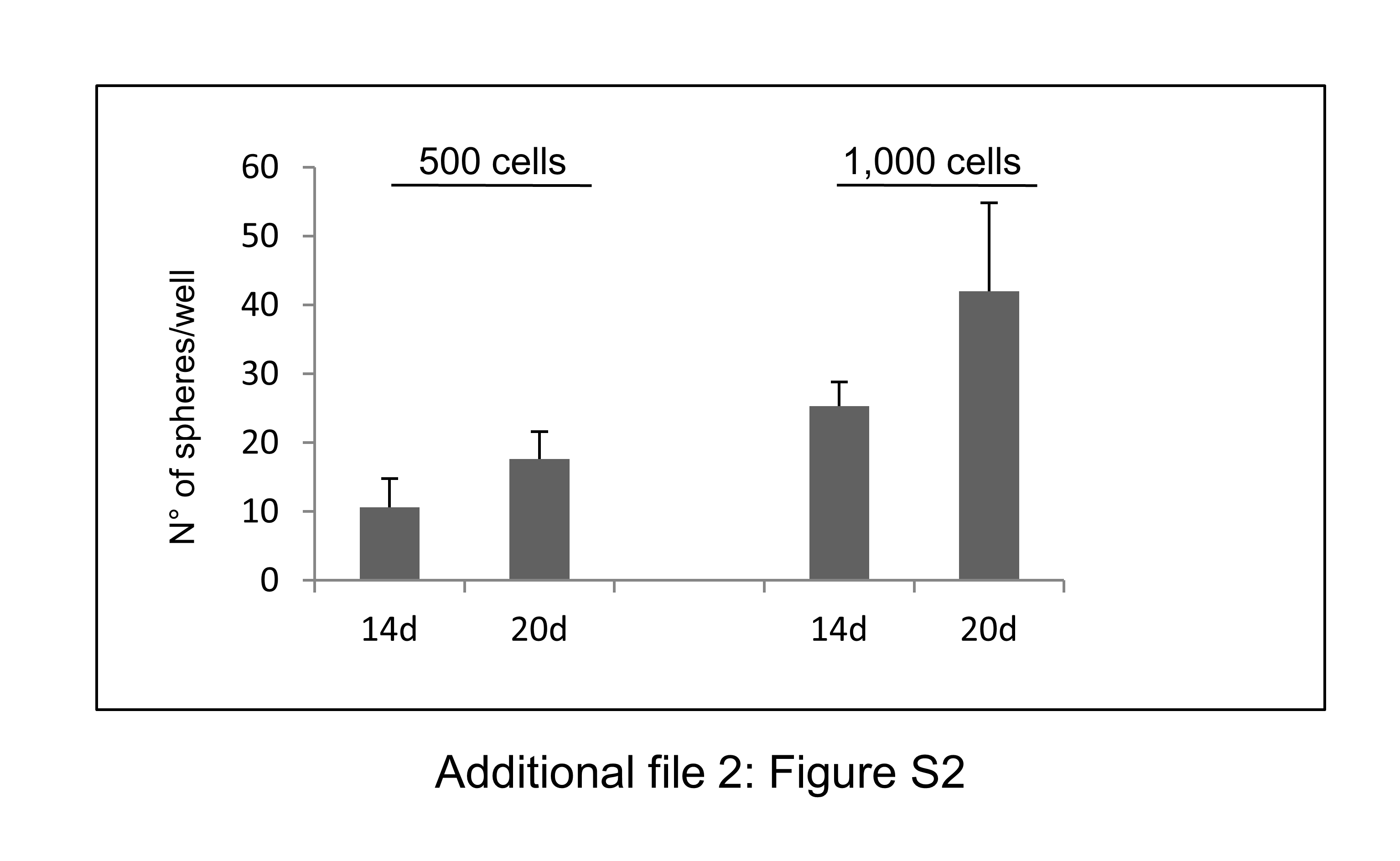

Supplement: Additional file 2: Figure S2. — Histogram of rhabdospheres formation dependent on number of cells. RD seeded at 500 or 1,000 cells/well in SC-medium (three replicates/sample). Rhabdosphere were counted at indicated times. The sphere numbers are displayed as mean ± S.D. (TIF 122 kb) [file 12943_2016_501_MOESM2_ESM.tif]

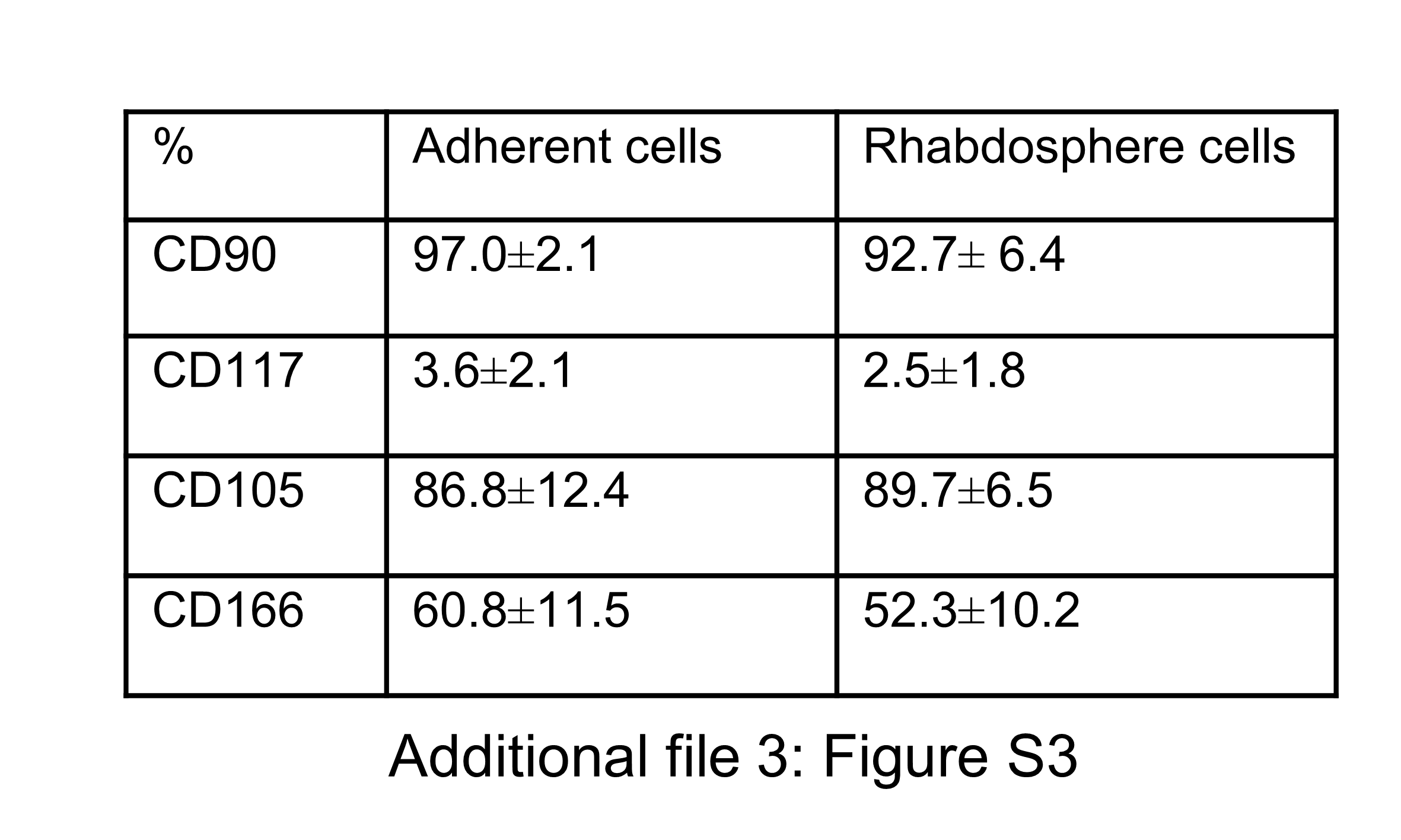

Supplement: Additional file 3: Figure S3. — Results of FACS analysis of the levels of CD90, CD117 and CD105 positive populations, which remain unchanged in SC-medium with respect to adherent condition. The percentage is displayed as mean ± S.D. of 3 experiments. (TIF 139 kb) [file 12943_2016_501_MOESM3_ESM.tif]

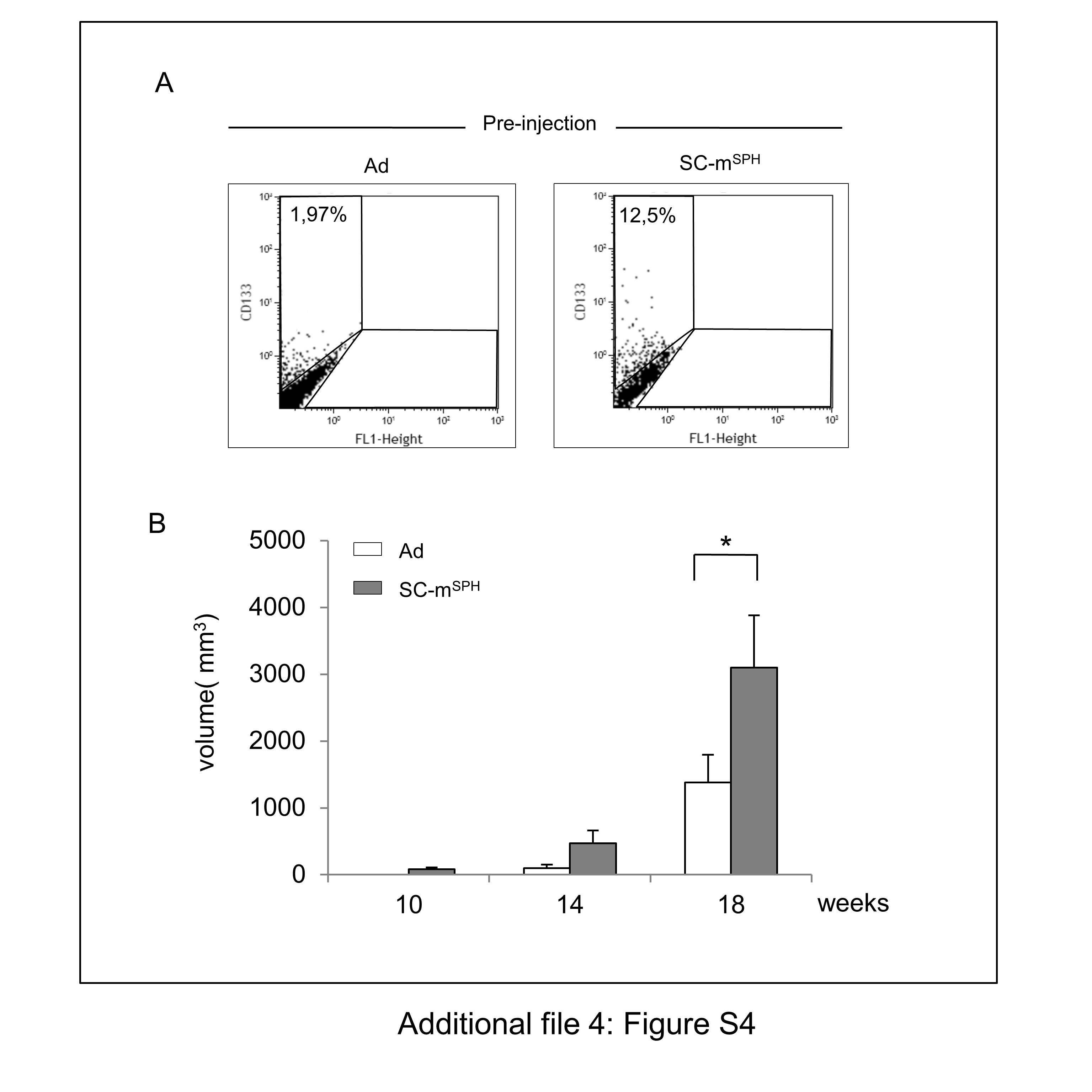

Supplement: Additional file 4: Figure S4. — Tumorigenic assay of rhabdosphere cells. A) FACS analysis of CD133 positive cells in adherent and in rhabdosphere cells (SC-mSPH) before subcoutaneously injection (pre-injection); B) histogram showing the tumor development and volumes of xenografts from adherent (Ad) or rhabdosphere cells (SC-mSPH), (n = 6, *p > 0,05). (TIF 341 kb) [file 12943_2016_501_MOESM4_ESM.tif]

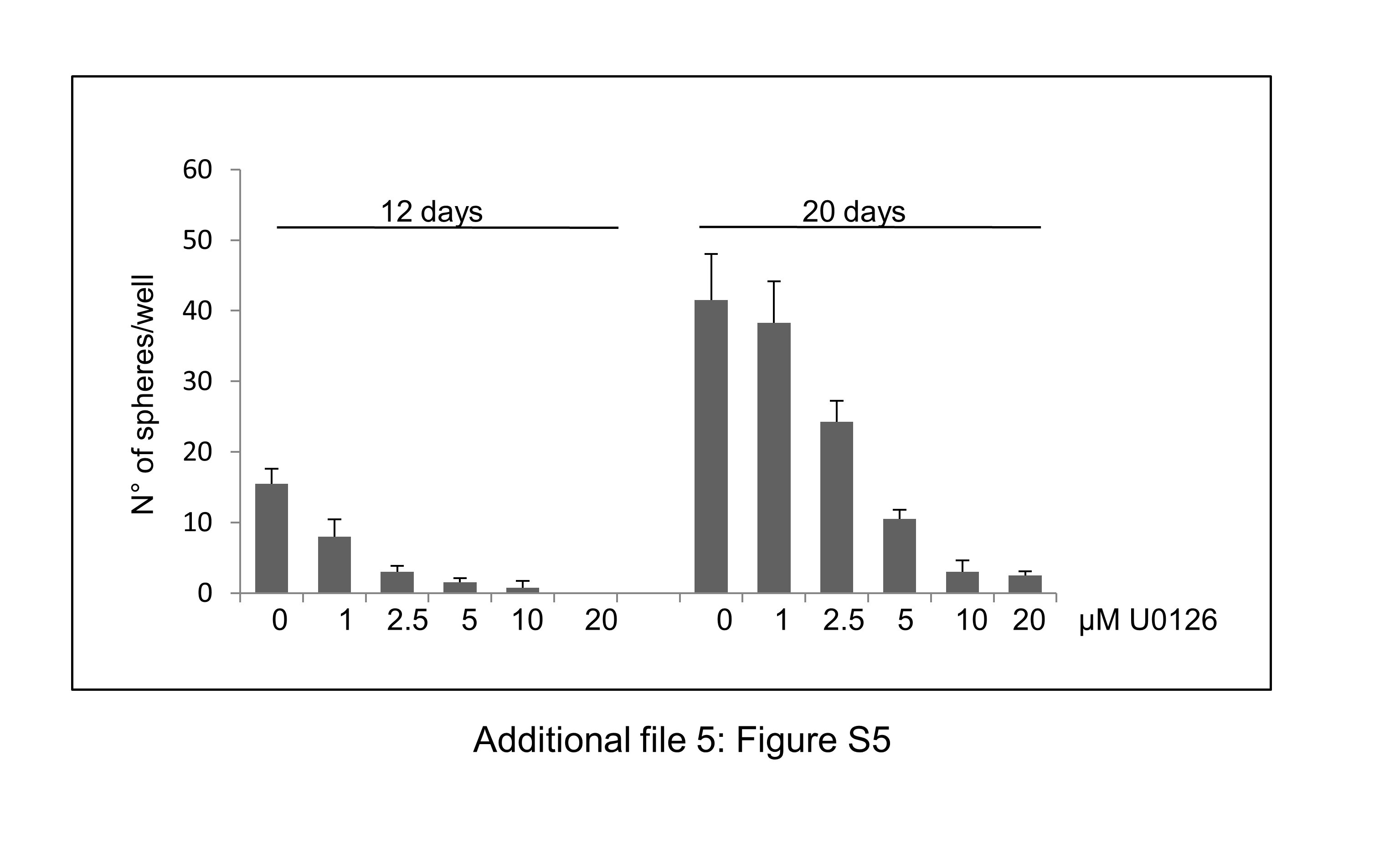

Supplement: Additional file 5: Figure S5. — U0126 dose/response of rhabdospheres inhibition. Histogram of two independent experiments of RD cells seeded in SC-medium (1,000 cells/well, three replicates per treatment) without or with indicated concentration of U0126. At13th day 1/3 of volume of fresh SC-medium containing the indicated U0126 concentration was added. Rhabdospheres were counted at indicated times. The sphere numbers are displayed as mean ± S.D. (TIF 122 kb) [file 12943_2016_501_MOESM5_ESM.tif]

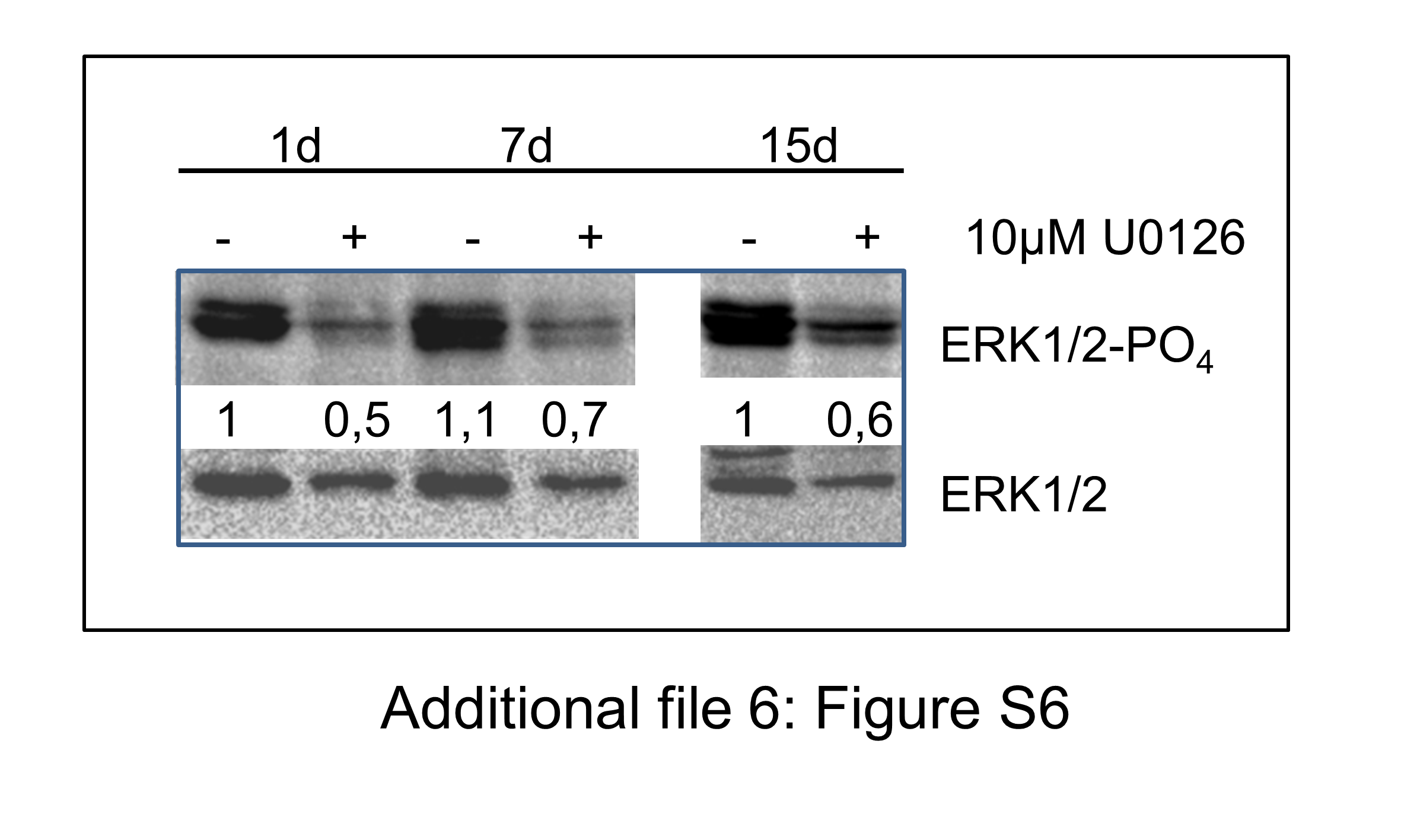

Supplement: Additional file 6: Figure S6. — Persistent phospho-ERK1/2 (ERK1/2-P) down regulation by U0126. Western blot analysis of protein lysates from RD in SC-medium without or with 10 μM U0126 at indicated times. The expression of ERK1/2-P was analysed and quantified by densitometry with respect to ERK/1/2. Representative experiment is shown. (TIF 385 kb) [file 12943_2016_501_MOESM6_ESM.tif]

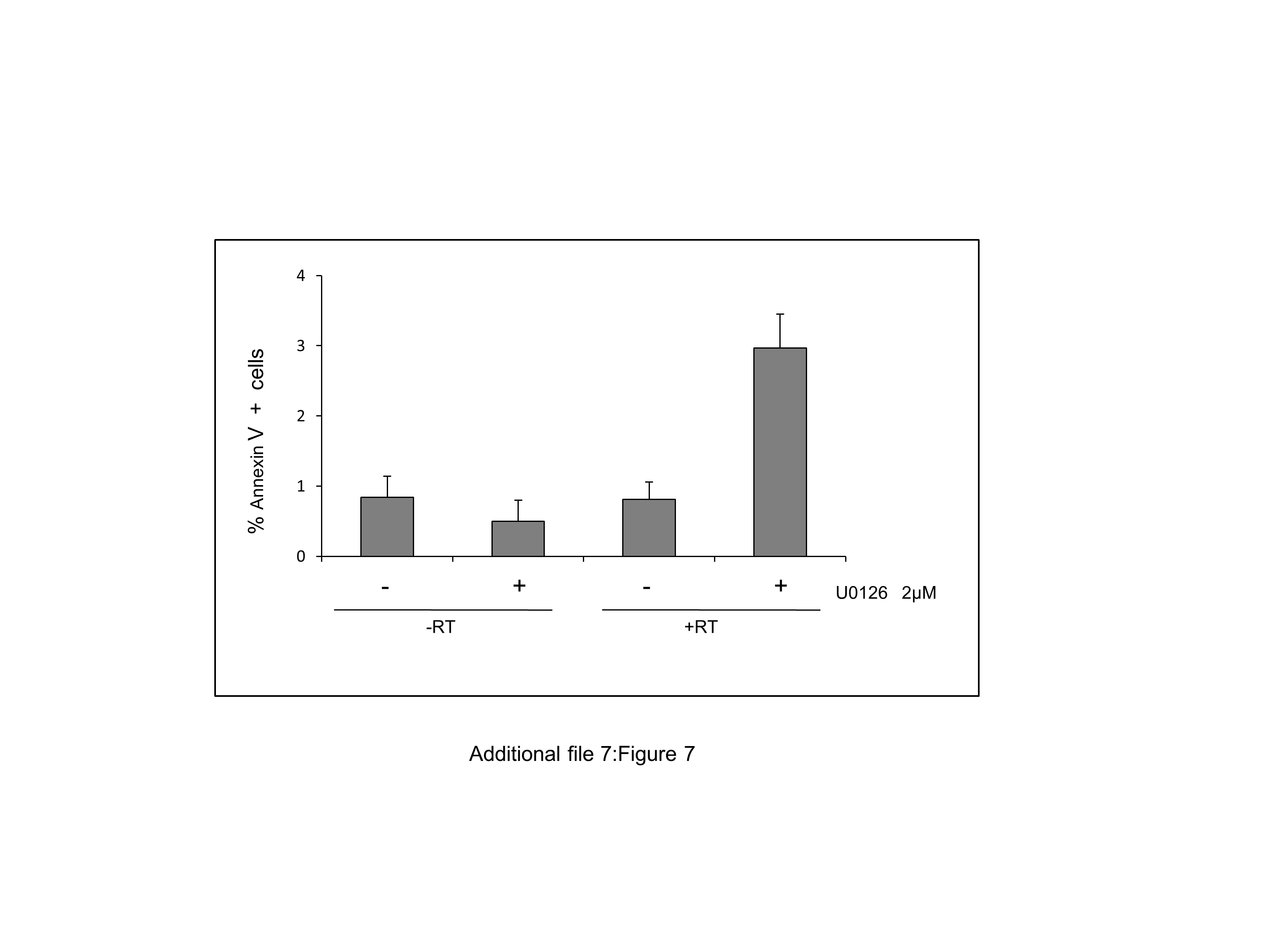

Supplement: Additional file 7: Figure S7. — Radiation (RT) or/and 2 μM U0126 were provided after RD rhabdophere spheres formation. Analysis of cell death by flow cytometric detection of Annexin-V staining of samples analyzed after 6 hours of treatment. (TIF 78 kb) [file 12943_2016_501_MOESM7_ESM.tif]
